# A Rapid and Accurate UHPLC Method for Determination of Monosaccharides in Polysaccharides of Different Sources of Radix Astragali and Its Immune Activity Analysis

**DOI:** 10.3390/molecules29102287

**Published:** 2024-05-13

**Authors:** Yali Guo, Lijun Wang, Kaishuang Liu, Meifang Li, Yibao Jin, Lifei Gu, Xie-An Yu, Shuhong Wang, Ping Wang, Bing Wang, Tiejie Wang

**Affiliations:** 1 NMPA Key Laboratory for Quality Research and Evaluation of Traditional Chinese Medicine, Shenzhen Institute for Drug Control, Shenzhen 518057, China; guoyaqwei2000@163.com (Y.G.);; 2 School of Pharmacy, Shenyang Pharmaceutical University, Shenyang 110016, China

**Keywords:** Radix astragali, monosaccharides, ultra-performance liquid chromatography, different sources, principal component analysis

## Abstract

With the escalating demand for Astragalus polysaccharides products developed from Radix Astragali (RA), the necessity for quality control of polysaccharides in RA has become increasingly urgent. In this study, a specific method for the simultaneous determination of seven monosaccharides in polysaccharides extracted from Radix Astragali (RA) has been developed and validated using ultra-performance liquid chromatography equipped with an ultraviolet detector (UHPLC-UV) for the first time. The 1-phenyl-3-methyl-5-pyrazolone (PMP) derivatizations were separated on a C18 column (Waters ACQUITY^TM^, Milfor, MA, USA, 1.8 µm, 2.1 × 100 mm) using gradient elution with a binary system of 5 mm ammonium formate (0.1% formic acid)-acetonitrile for 24 min. Additionally, seven monosaccharides showed good linear relationships (R^2^, 0.9971–0.9995), adequate precision (RSD < 4.21%), and high recoveries (RSD < 4.70%). The established method was used to analyze 109 batches of RA. Results showed that the Astragalus polysaccharides (APSs) mainly consist of mannose (Man), rhamnose (Rha), glucose (Glu), galactose (Gal), arabinose (Ara), xylose (Xyl); and fucose (Fuc); however, their composition was different among RA samples from different growth patterns, species, growth years, and origins, and the growth mode of RA and the age of wild-simulated RA can be accurately distinguished by principal component analysis (PCA). In addition, the immunological activity of APSs were also evaluated jointly by measurement of the NO release with RAW264.7, with the results showing that APSs have a promoting effect on the release of NO and exhibit a significant correlation with Man, Glu, Xyl, and Fuc contents. Accordingly, the new established monosaccharides analytical method and APS-immune activity determination in this study can provide a reference for quality evaluation and the establishment of quality standards for RA.

## 1. Introduction

Radix Astragali (RA), is a dried root of the leguminous plant *Astragalus membranaceus* (Fisch.) Bge. var. *mongholicus* (Bge.) Hsiao or *Astragalus membranaceus* (Fisch.) Bge [1]. It is a commonly used traditional Chinese medicinal herb in China and throughout the world [2]. It was first recorded in the “Shennong Materia Medica Classic” and has been in use for over 2000 years, being known as the “most nourishing medicine” [3]. Clinical studies have shown that RA possesses various biological activities such as enhancing the body’s immunity, as well as anti-tumor and antivirus properties [4,5]. RA contains rich nutritional components, and its main chemical components include saponins, flavonoids, polysaccharides, amino acids, and other components [6]. Among them, saponins, flavonoids, and polysaccharides are widely recognized as active ingredients of RA, and various substances, such as Astragaloside I, II, III, IV, Calycosin-7-O-beta-D-glucoside, Ononin, and Astragalus polysaccharides (APSs), have been proven to have strong biological activities, such as immunomodulatory, hypoglycemic, antioxidant, and liver protective effects [7,8]. In addition, there are many preparations containing RA (RA essence, RA granules, RA donkey-hide gelatin oral liquid, RA injection, Yiqi Yangxue oral liquid, etc.) in clinics that are used to nourish blood and “qi” (“qi” in traditional Chinese medicine is one of the fundamental substances that make up the human body and maintain life activities) [9].

APSs are the most abundant substances in RA [10]. Moreover, studies have shown that APSs have various biological activities such as enhancing immunity, anti-tumor, and delaying aging [11,12,13]. In order to establish a holographic quality evaluation method of RA, APSs should also be used as an important index for quality evaluation of RA. In addition, the quality and biological activity of RA are affected by many factors, such as growth patterns, species, growth years, and origins, which may be the key factors affecting its quality [14,15,16]. However, to the best of our knowledge, the effects of these factors on its polysaccharides and immune activity have not been comprehensively studied. Furthermore, the biological activities of polysaccharides are affected by their various structural properties, which are closely related to the monosaccharides chain sequence and branching structure [17]. Furthermore, it has been reported that different monosaccharide compositions often result in polysaccharides possessing different biological activities. For example, polysaccharides with radiation protection activity are often rich in galacturonic acid, galactose, and arabinose [18]. In addition, the monosaccharide composition of polysaccharides with intestinal barrier protection function is galactose (Gal), mannose (Man), arabinose (Ara), xylose (Xyl), and rhamnose (Rha) [19]. Similarly, high levels of uronic acid exhibit good liver protective activity [20]. Therefore, it is very necessary to establish a sensitive and rapid monosaccharides determination method in order to further study the quality of the polysaccharide of RA from different sources. This will provide important theoretical value for the comprehensive and effective development and utilization of RA resources.

At present, the main methods for the detection of monosaccharides in polysaccharides include HPLC, GC and UHPLC-MS [21,22,23]. Among them, the HPLC method is commonly used for analysis, but its analysis time is generally long. Ultra-performance liquid chromatography (UHPLC) has evolved from HPLC. Compared to HPLC, the key advantages of UHPLC are faster analysis speed, higher separation efficiency, better peak shape, less eluent consumption, and higher sensitivity, which have made UHPLC a preferred option for scientists. However, the feasibility of using UHPLC-UV for the quantification of monosaccharides has scarcely been studied. Therefore, in our research, a specific method for simultaneous determining of seven monosaccharides of polysaccharides in RA has been developed and validated by UHPLC-UV. The optimization of polysaccharides hydrolysis conditions and the 1-phenyl-3-methyl-5-pyrazolone (PMP) precolumn derivatization conditions have also been comprehensively studied. After that, the newly developed method was successfully applied to analyze the APSs from 109 batches of RA. Meanwhile, the immunological activities of APSs were also evaluated jointly by measurement of the NO release with RAW264.7, which were further used for the estimation of structure-activity relationships. In summary, this provides a reference for the quality screening and resource development of RA, laying a foundation for improving its quality evaluation system.

## 2. Results and Discussion

### 2.1. Information on the Collected Herb RA

A total of 109 batches of typical representative RA samples with different growth patterns, species, growth years, and origins were collected from January 2021 to August 2022. All samples were authenticated by Prof. Zhang Ji, the director of the Chinese Medicine Specimen Library of the former National Institutes for Food and Drug Control. Detailed information about the RA samples is provided in Table 1.

### 2.2. Method Validation

#### 2.2.1. Linearity, LOD and LOQ

The calibration curves were constructed by plotting the peak area versus the concentration of the corresponding standard solutions. Table 2 shows the summary of calibration curves and linear ranges. The linear range of each monosaccharide was determined based on the content in the RA sample, and all calibration curves showed good linearities (R^2^ ≥ 0.9971) within the test ranges. Furthermore, the limit of detection (LOD) and limit of quantification (LOQ) of each analyte were determined at S/Ns (signal-to-noise ratios) of approximately 3 and 10, respectively. The results showed that the LOD and the LOQ were in the ranges of 0.66–25.67 and 0.97–38.63 μg/mL on the column, respectively, which is higher than the values obtained by the UHPLC-PDA methods for the analysis of *Coriolus versicolor* Polysaccharides [24]. However, the sensitivity of this method is adequate for the purpose of APS analysis.

#### 2.2.2. Precision

The measurement of intra-day and inter-day variations were used to evaluate the precision of this method. Seven mixed monosaccharide standard solutions, including Man, Rha, Glu, Gal, Ara, Xyl and Fuc, were determined under set UHPLC conditions at the concentrations of 0.0792 mg/mL, 0.102 mg/mL, 5.133 mg/mL, 0.404 mg/mL, 0.1812 mg/mL, 0.0308 mg/mL, 0.0492 mg/mL, respectively. The relative standard deviation (RSD) of peak area were used as the criteria for the validation. As shown in Table 3, the RSD of intra-day and inter-day precision were less than 2.48% and 4.21%, respectively, indicating that the instrument precision was satisfactory.

#### 2.2.3. Recovery

Monosaccharide standard solutions, including Man, Rha, Glu, Gal, Ara, Xyl and Fuc, were added after acid hydrolysis at the corresponding concentration (0.0719 mg/mL, 0.0646 mg/mL, 7.18 mg/mL, 0.448 mg/mL, 0.460 mg/mL, 0.0405 mg/mL, 0.0178 mg/mL, respectively) of the same monosaccharide in the sample (*n* = 6). The recovery rate was calculated as follows: spike recovery(%) = (found amount − original amount) × 100%/spiked amount. This accuracy was within the acceptable ranges (Table 3).

#### 2.2.4. Repeatability

Six different solutions prepared under the same conditions were used to assess the repeatability. The results demonstrated that the RSD values were all less than 3.77% (Table 3), indicating that the method had excellent repeatability.

#### 2.2.5. Stability

Analysis of the same sample solution six times at different times (0, 2, 4, 6, 8, 10, 12 and 24 h) was used to assess its stability. The RSD values of the stability were less than 4.04% (Table 3), indicating that the sample solution was stable within 24 h.

### 2.3. Optimization of Acid Hydrolysis of the APS

**Figure 1:**
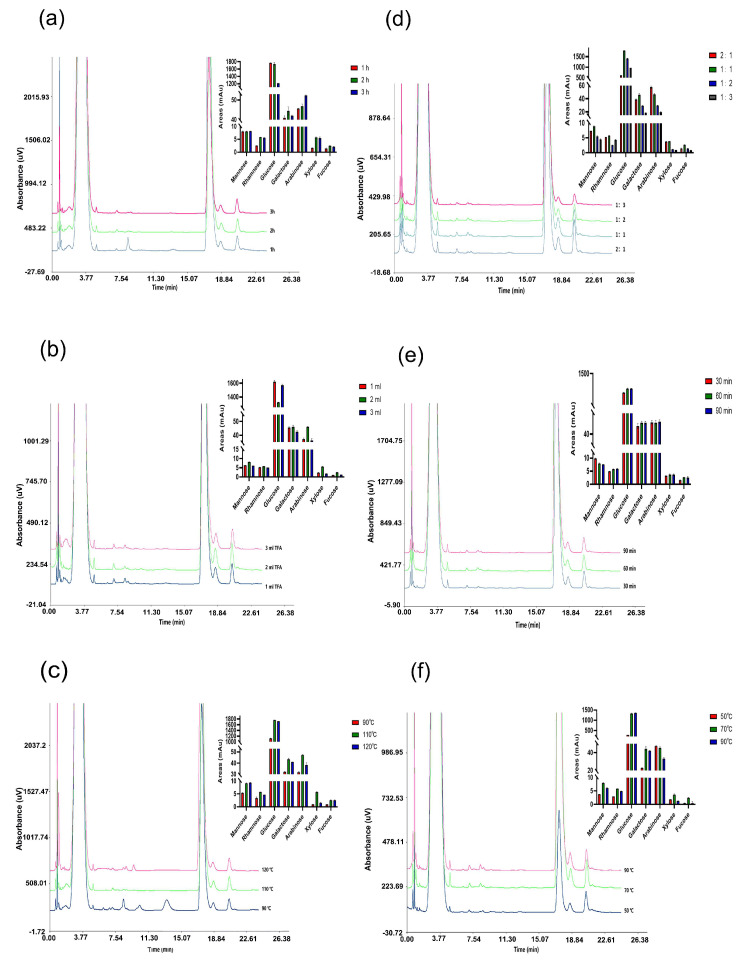
Chromatogram and monosaccharide peak area of polysaccharide samples under different hydrolysis conditions (**a**) different acid hydrolysis time, (**b**) different amounts of TFA, (**c**) different acid hydrolysis temperature and derivatization conditions (**d**) different proportions of PMP and samples, (**e**) different derivatization time, (**f**) different derivatization temperature.

The optimization of hydrolysis condition is necessary for the exact quantitative analysis of the APS. As shown in Figure 1a, the effect of hydrolysis time was firstly investigated. The amounts of almost all monosaccharides peaked when the hydrolysis time was 2 h and then decreased with time. In addition, the amount of Trifluoroacetic Acid (TFA) played a critical role in the hydrolysis process, and the results indicated that polysaccharide cannot be completely hydrolyzed with 1.0 mL TFA. When the amount of TFA solution was 2 mL, the monosaccharide content was highest. The effects of temperature was shown in Figure 1b. The release of all monosaccharides initially increased as the temperature increased and went through a peak (110 °C) and then decreased (Figure 1c). Therefore, the optimum hydrolysis condition was hydrolysis time 2 h, 2 mL TFA solution and temperature 110 °C. 

### 2.4. Optimization of Precolumn Derivatization of the Hydrolysates of the APS

As shown in Figure 1d, the amount of sample was a key factor affecting the amounts of PMP-derivatives, and the volume ratio of sample to PMP solution was 2:1, while the derivatization was incomplete. When the ratio reached 1:1, the chromatographic peak area of the PMP-derivatives was the highest. The effect of time on the derivatization of PMP and monosaccharide was also investigated. The results showed that the amounts of PMP-derivatives increased as the time increased, while the amounts of PMP-derivatives did not change with time after 60 min (Figure 1e). In addition, Figure 1f revealed that an excessively high reaction temperature might be detrimental to the stabilities of some derivatives. Therefore, the optimum derivatization condition was: sample: derivative reagent = 1:1, derivatization time 60 min, derivatization temperature 70 °C.

### 2.5. Determination of Monosaccharide Composition and Content in APSs

#### 2.5.1. Establishment of UHPLC Fingerprint

**Figure 2:**
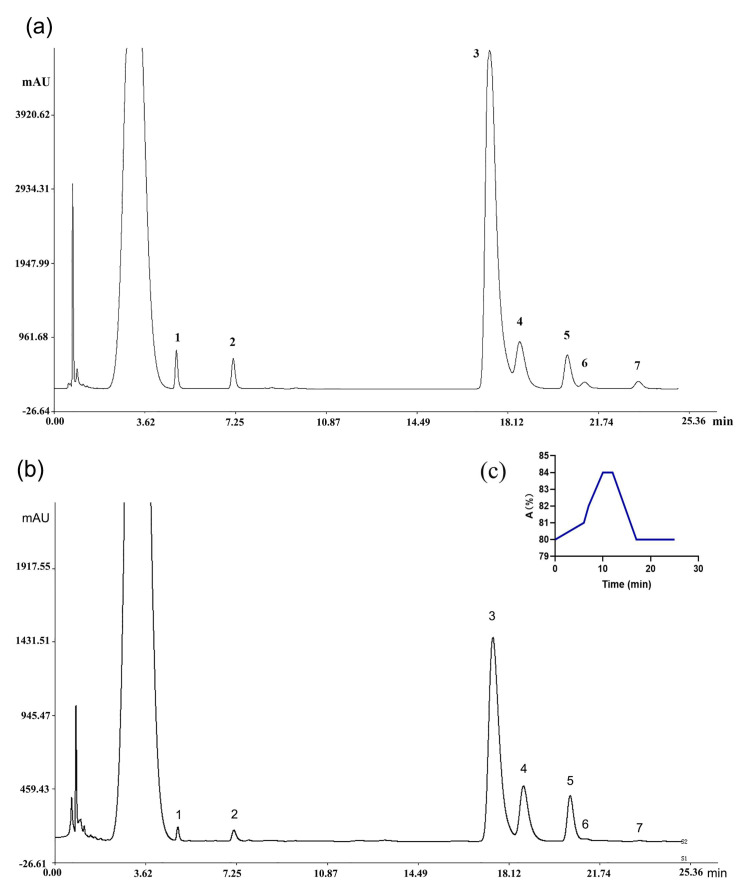
PMP-derivative chromatogram of (**a**) monosaccharide standard and (**b**) sample; (**c**) the gradient profile. 1, Mannose; 2, Rhamnose; 3, Glucose; 4, Galactosee; 5, Arabinose; 6, Xylose; 7, Fucose. The band that appears at retention time 3.77 min is the chromatographic peak of residual PMP.

In this study, the established method was applied to determine the monosaccharide composition of 109 batches of RA samples. Figure 2 showed the UHPLC chromatogram of mixed monosaccharides standards (Figure 2a) and those in RA polysaccharide sample (Figure 2b). The UHPLC spectrum of 109 batches of samples imported into the Chromatographic Fingerprint of Traditional Chinese Medicine (Version 2004A, Chinese Pharmacopoeia Committee) and seven common peaks was identified.

#### 2.5.2. Similarity Evaluation 

The similarity values of samples from different growth patterns, species, growth years, and origins of RA samples were calculated using the similarity evaluation system (Table 4). For RA with different growth patterns, the similarity of 41 batches of wild-simulated RA samples ranged from 0.69 to 1.00. The relatively large variation in similarity reflects the impact of environmental differences, such as light, water, and soil, brought about by the wild-simulated cultivation method on the quality of APS. Using the UHPLC fingerprint of wild-simulated RA as a reference, the similarity of 68 batches of cultivated RA samples ranged from 0.84 to 1.00, indicating that the cultivation method ensured a certain degree of stability and homogeneity in the quality of RA. For RA with different origins, the similarity among 90 batches of MG samples ranged from 0.70 to 1.00, showing a relatively large variation. It can be preliminarily inferred that growth patterns, years, and origin have a significant impact on the APSs in MG. The overall chemical profiles of 19 batches of MJ samples were relatively similar, with a similarity to MG ranging from 0.96 to 1.00. For RA with different growth years, the 22 batches of 2-year-old cultivated RA exhibited high similarity, ranging from 0.95 to 1.00, indicating that the quality of APSs in 2-year-old cultivated RA was stable. Compared to the 2-year-old, the similarity of 20 batches of 3-year-old cultivated RA ranged from 0.74 to 1.00, suggesting that the growth years had a significant effect on APS. Additionally, the internal similarity varied greatly, making it difficult to accurately distinguish between 2-year-old and 3-year-old cultivated RA based on monosaccharide fingerprint similarity. For the 16 batches of wild-simulated RA with growth years of 5 years or less, the similarity ranged from 0.44 to 0.83. Furthermore, the similarity between these and 25 batches of wild-simulated RA with growth years exceeding 5 years ranged from 0.35 to 1.00, further suggesting that the growth years had a considerable impact on APSs. A similarity analysis of UHPLC spectra from four production areas showed that the monosaccharide composition of APSs in RA from the Gansu and Shanxi provinces had high similarity, which implies similar quality between them. 

#### 2.5.3. Monosaccharide Content of RA from Different SOURCES

According to linear equation, the molar amount and molar ratio of each monosaccharide were calculated (Tables S1 and S2). As shown in Table 5, APSs from different sources contained similar monosaccharide compositions of Man, Rha, Glu, Gal, Ara, Xyl, and Fuc, with their average molar ratio being 0.52:0.66:87.89:5.30:5.18:0.33:0.11. It was obvious that Glu was the major sugar in APS, ranging from 0.46 to 4.48 mmol/g, followed by Gal, with a content between 0.02 and 0.27 mmol/g. The lowest content was Fuc, between 0.0005~0.01 mmol/g (Table S1). Additionally, the contents and ratio of these monosaccharides of APSs from different sources were different.

- Analysis of RA with Different Growth Patterns

**Figure 3:**
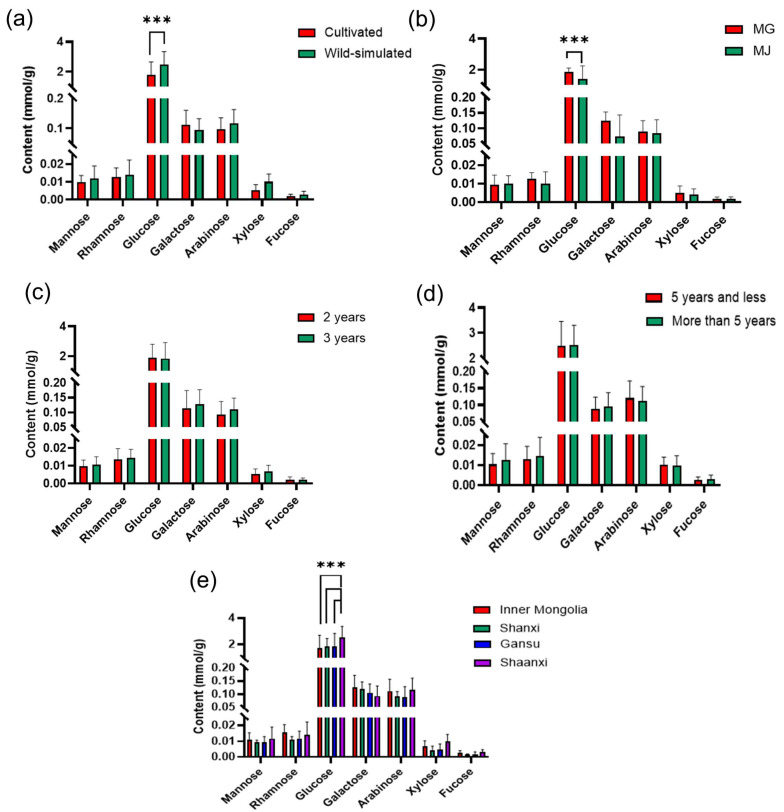
Comparison of the molar amounts of monosaccharides in APSs of RA from (**a**) different growth patterns, (**b**) different species (MG (*Astragalus membranaceus*. var. *mongholicus*) and MJ (*Astragalus membranaceus*)), different growth years of (**c**) cultivate RA aged 2 and 3 years and (**d**) wild-simulated divided by 5 years, (**e**) different origins ( *** *p* < 0.001).

**Figure 4:**
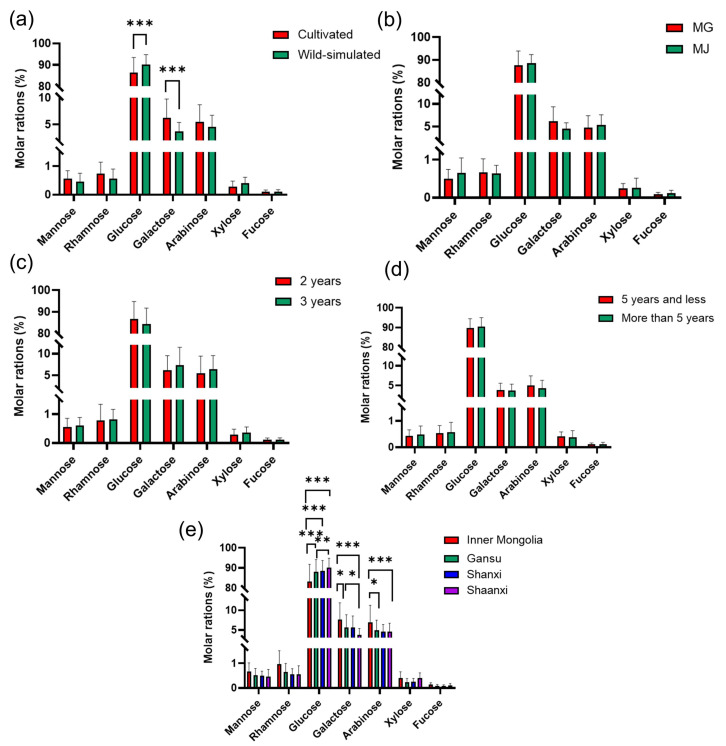
Comparison of the molar rations of monosaccharides in APSs of RA from (**a**) different growth patterns, (**b**) different specie, different growth years of (**c**) cultivate RA aged 2 and 3 years and (**d**) wild-simulated divided by 5 years, (**e**) different origins (* *p* < 0.05, ** *p* < 0.01, *** *p* < 0.001).

As shown in Figure 3a and Figure 4a, there was a significant difference in the content of Glu and Gal between wild-simulated RA and cultivated RA. The molar ratio of Gal in wild-simulated RA was significantly higher than that of cultivated RA. The molar amount and molar ratio of Glu in wild-simulated RA (2.49 mmol/g and 90.2%) were higher than those of cultivated RA (1.80 mmol/g and 86.5%). Therefore, the growth patterns can be preliminarily inferred by measuring the content of Glu. In addition, from the molar ratio, it can be seen that the cultivated pattern may be beneficial to the accumulation of five monosaccharide components, such as Man, Rha, Gal, Ara and Fuc, but not of Glu and Xyl.

- Analysis of RA with Different Species

The controversy over the classification of Mongolian Astragalus (MG) and membranous Astragalus (MJ) has been ongoing for decades [25]. Due to the crucial role of traditional Chinese medicine identification in pharmacology and clinical administration, it is necessary to conduct a differential analysis between MG and MJ. The results of Figure 3b and Figure 4b show that, except for the lower molar ratio of Glu (85.8%) in MG compared to MJ (88.2%), the monosaccharide content was higher than that of MJ.

- Analysis of RA with Different Growth Years

The 2-year-old cultivated RA is the main commodity circulating in the market [26]. Among the cultivated RA, the molar content of each monosaccharide in the APS of the 2-year-old and 3-year-old (including 2.5 years, 3 years, and 3.5 years) samples was shown in Figure 3c and Figure 4c. The content of Glu in the 3-year-old RA was significantly different from that of the 2-year-old RA, and its molar amount was higher than that of the 2-year-old, but the molar ratio content was the opposite. Furthermore, the content of other monosaccharides in the 3-year-old RA was generally higher than that of the 2-year-old RA. In addition, the People’s Republic of China’s group standard “Commercial Specifications and Grades of Chinese Medicinal Materials-RA” stipulates that the age of wild-simulated RA should be over 5 years [27]. Therefore, the difference between APSs with a cutoff of 5 years was analyzed. As shown in Figure 3d and Figure 4d, there was no significant difference in the monosaccharide content of RA samples above and below 5 years. It was speculated that the change in monosaccharide content of samples of different ages was not a simple increase or decrease with the increase in age.

- Analysis of RA with Different Origins

China has a vast territory, and due to different geographical locations, there are significant environmental differences in different regions, which will affect the chemical composition of RA [14]. The comparison of monosaccharides in the APSs of different regions is shown in Figure 3e and Figure 4e. The contents of Glu, Gal, and Ara variy greatly among the four regions. The experimental results showed that there were significant differences in molar amount and molar ratio of Glu among Inner Mongolia (1.73 mmol/g and 83.2%), Gansu (1.84 mmol/g and 87.9%), Shanxi (1.86 mmol/g and 88.4%), and Shaanxi (2.51 mmol/g and 90.2%). In addition, the molar ratios of Man, Rha, Gal, Ara, Xyl, and Fuc in Inner Mongolia were the highest, while Glu was the lowest among the samples of four different regions (Figure 4e). However, there was little difference in the content of monosaccharides in Shanxi and Gansu. 

In summary, the differences between RA from different sources can provide a theoretical basis and technical support for the evaluation of RA product quality. 

#### 2.5.4. Chemical Pattern Recognition Analysis

**Figure 5:**
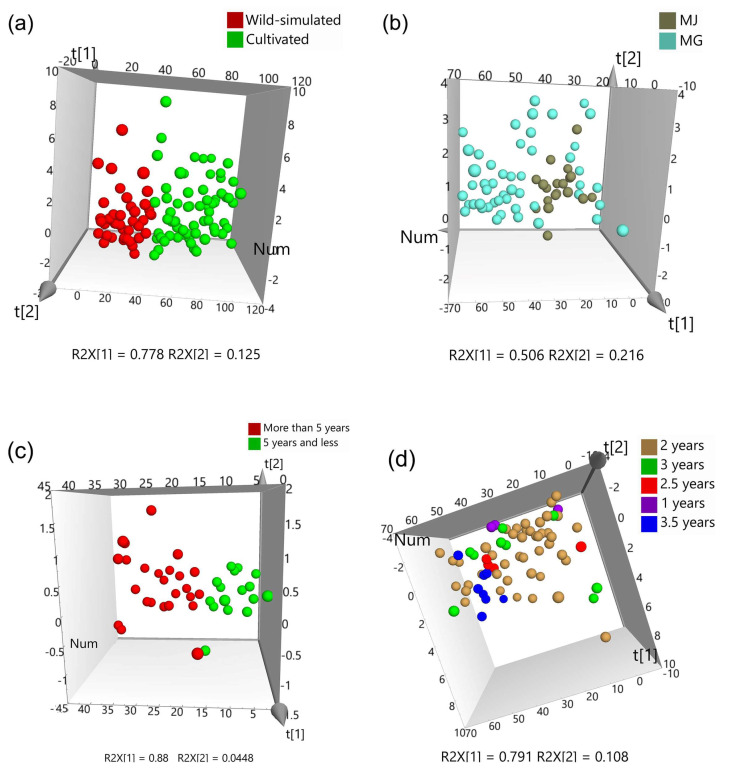
PCA analysis of RA samples from (**a**) different growth patterns, (**b**) different species, different growth years of (**c**) wild-simulated and (**d**) cultivate.

Principal component analysis (PCA) is an unsupervised dimensionality reduction data processing method that can truly reflect the grouping of the original data [28]. In this study, PCA analysis was performed by SIMCA 14.1 software using the mole ratio of seven monosaccharides in 109 batches of RA samples as the data matrix. The scatter plot of RA of two growth patterns was shown in Figure 5a. The variance contribution rate of the first principal component was 77.8%, and the cumulative contribution rate of the first two principal components exceeded 90%, which can objectively reflect most of the information of the APSs. From the scatter plot, it can be seen that there was a clear boundary between the RA from two growth patterns, and the distribution of each sample was closely clustered, indicating that the acquisition method was stable and reliable. Additionally, the PCA method can be used to distinguish growth patterns by determining the monosaccharide composition of APSs. 

The classification results of species was shown in Figure 5b, indicating that the distribution of MJ samples was relatively concentrated, with little difference; however, MG was more dispersed, with greater differences. Therefore, MG and MJ cannot be distinguished based on the monosaccharide composition of APS. 

Due to the fact that wild-simulated RA for more than 5 years was the “true” RA, we analyzed wild-simulated RA for both 5 years and less and for more than 5 years. The results in Figure 5c show that the cumulative contribution rate of the first two principal components exceeds 90%, while the classification accuracy rate was 100%, indicating that the PCA method can be used to determine whether the growth years of wild-simulated RA exceeds 5 years by measuring the monosaccharide composition of its polysaccharide.

In addition, the 2-year cultivated RA, which was the mainstream in the market, was classified from RA with other growth years. As shown in Figure 5d, although there was some overlap between samples of different growth years, there was internal clustering, indicating that there were certain differences in the monosaccharide composition of APSs with different growth years.

### 2.6. Immunomodulatory Activity of APS

**Figure 6:**
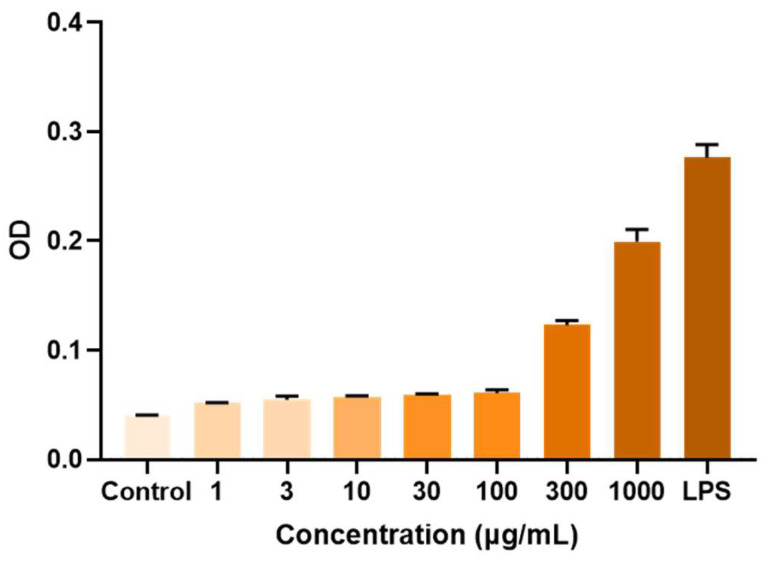
Effect of APSs of the different concentration on the release of NO by macrophages.

**Figure 7:**
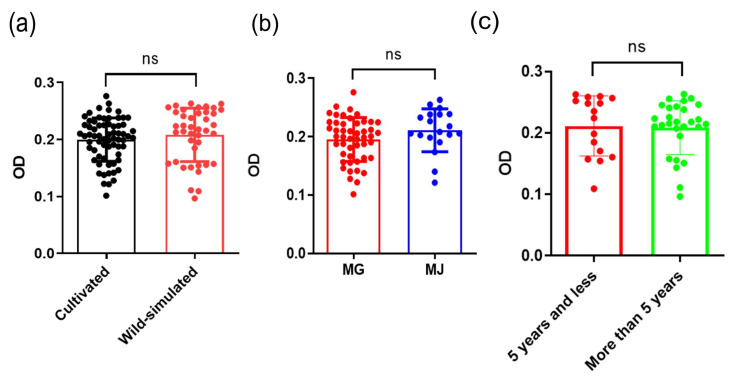
Effect of APSs from (**a**) different growth patterns, (**b**) different species and (**c**) different growth years of wild-simulated on the release of NO by macrophages, “ns” means “not significant”.

**Figure 8:**
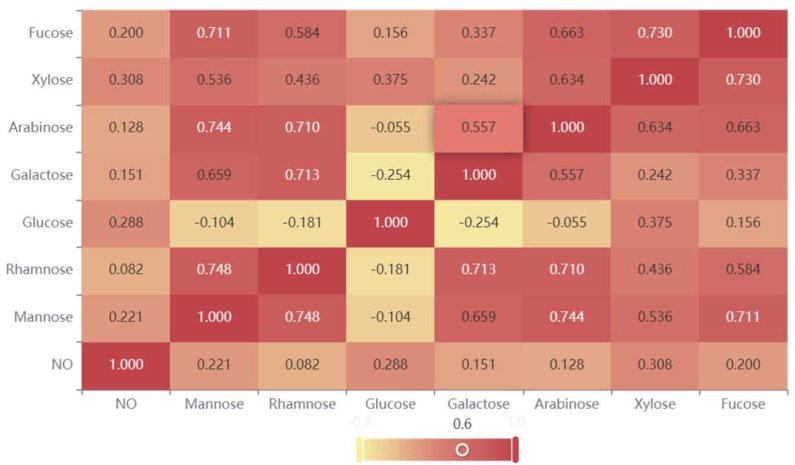
Spearman correlation coefficient heat map.

When stimulated by external stimuli, stress cells such as macrophages release Nitric Oxide (NO), which serves as a signaling molecule involved in signal transduction in the body and plays a role in killing or inhibiting pathogen reproduction in the immune system [29]. At mass concentrations of 30, 100, 300, 1000 μg/mL of APSs, or 1 ug/mL of LPS that were treated on macrophages RAW264.7 for 24 h, the NO production released by macrophages was significantly higher than that of the normal control group. APSs could stimulate the NO production in a dose-dependent manner. The higher the concentration of APS, the greater the NO production (Figure 6). As shown in Figure 7, there was no significant difference in the effect of RA from different sources on NO release, although they were clearly separated in the PCA model. In addition, the Spearman correlation analysis results were shown in Figure 8, indicating a significant correlation with Man, Glu, Xyl, and Fuc. Therefore, it can be speculated that the release of NO by macrophages was the result of the joint influence of multiple monosaccharides.

## 3. Materials and Methods

### 3.1. Chemicals, Reagents and Materials

Fuc, Rha, Ara, Xyl, Man, Glu, Gal were purchased from the National Institutes for Food and Drug Control (Beijing, China). Trifluoroacetic acid (TFA) and 1-phenyl-3-methyl-5-pyrazolone (PMP) were purchased from Shanghai Aladdin Biochemical Technology Co., Ltd. (Shanghai, China). Fetal bovine serum (FBS, Gibco, Waltham, MA, USA) and DMEM media (Gibco, Grand Island, NY, USA) were used for cell culture. Nitric oxide detection kits were purchased from the Beyotime Institute of Biotechnology (Shanghai, China). Milli-Q^®^ water was purified in-house by a Milli-Q Academic ultrapure water system (Millipore, Milford, MA, USA). Other chemical reagents were analytical grade.

### 3.2. Preparation of Sample Solutions and Standard Solutions

#### 3.2.1. Preparation of Standard Solution

Monosaccharide standards, including Man, Rha, Glu, Gal, Ara, Xyl and Fuc, were accurately weighed and dissolved in water to obtain the mixed solution at concentrations of 0.396 mg/mL, 0.51 mg/mL, 25.665 mg/mL, 2.02 mg/mL, 0.906 mg/mL, 0.154 mg/mL, 0.246 mg/mL, respectively.

#### 3.2.2. Extraction of APS

The RA samples were dried in a 50 °C blast drying oven for 1 h, crushed, and passed through a No. 4 sieve for further use. The RA sample powder was precisely weighed and mixed with pure water according to the material-liquid ratio of 1:30 g/mL. It was ultrasonicated twice at 55 °C for 20 min (power 80 W). The mixture was centrifuged, and then the supernatant was concentrated at 55 °C under reduced pressure. Then, ethanol was added to the extract until its final concentration reached 80% (*v*/*v*), and the mixture was allowed to sit overnight at 4 °C. The precipitates were collected by centrifugation (5000 rpm for 10 min), dissolved in appropriate water, and then freeze-dried to obtain APSs.

#### 3.2.3. Monosaccharide Composition Analysis of APS

An amount of 10 mg of an APS sample was dissolved in 2 mL trifluoroacetic acid (2 mol/L, TFA) in a sealed glass tube, then placed in an air-drying oven to hydrolyze into component monosaccharides at 110 °C for 2 h. After the glass tube was cooled to room temperature, the TFA was evaporated using a nitrogen blowing instrument. To completely remove the remaining TFA, the same amount of methanol was added and dried. The procedure was repeated 3 times. After that, the residue was re-dissolved in 0.5 mL of distilled water to obtain the APS hydrolysate. Then, 0.30 mL of the above solution, 0.30 mL of PMP methanol solution (0.5 mol/L), and 0.30 mL of NaOH solution (0.3 mol/L) were added to a test tube with a stopper, mixed well, and put into a constant temperature water bath at 70 °C for 60 min, followed with 0.30 mL of HCl (0.3 mol/L) for neutralization. The aqueous layer was filtered by a 0.22 μm pore membrane filter for UHPLC analysis. The same derivatized method was performed with the standard monosaccharide mixed solution.

### 3.3. Instrumentation and Chromatographic Conditions

The monosaccharide derivatives were analyzed by UHPLC (Thermo Ultimate300 BioRS, Waltham, MA, USA), which was equipped with a quaternary solvent delivery system and a DAD detector. Analytes were separated on a C18 column (ACQUITYTM Premier HSS T3, 1.8 µm, 2.1 × 100 mm) and the column temperature was kept at 30 °C. The mobile phase flow rate was 0.4 mL/min, and the injected volume was 3 μL. The detection wavelength was 250 nm. Chromatography was performed using a step gradient (A: aqueous solution of 5 mM ammonium formate—0.1% formic acid, B: acetonitrile) and a gradient elution system program: 0–6 min, 80–81% A; 6–7 min, 81–82% A; 7–10 min, 82–84%; 10–12 min, 84–84%; 12–17 min, 84–80%; 17–25 min, 80–80%.

### 3.4. Nitric Oxide Release

A 100 μL suspension of RAW 264.7 cells were plated at 1 × 10^6^ cells/mL in 96-well plates and incubated for 24 h. After removing the medium, macrophages were firstly stimulated with different concentrations of APSs (1, 3, 10, 30, 100, 300, 1000 μg/mL) for 24 h to determine the proper concentration. In subsequent experiments, the macrophages were incubated with 300 μg/mL APS, 1 μg/mL LPS (as the positive group), or DMEM (as the control group). After 24 h, 50 μL of the supernatants were collected and reacted with Griess reagent at room temperature for 10 min. Nitrite products were determined by measuring absorbance at 540 nm with a microplate reader, and the average absorbance value of the 3 replicates was taken.

### 3.5. Data Processing

Data were processed by Graphpad Prism 9.0.0 software, using a two-tailed Student’s *t*-test and one-way analysis of variance (ANOVA). A *p* value less than 0.05 was considered statistically significant.

## 4. Conclusions

In this study, a UHPLC method for detecting seven kinds of monosaccharides was established, and the concentration levels detected by the UV detector equipped with UHPLC instrument proved sufficient for the study of RA samples. Compared to MS (mass spectrometry) detection, UV detection equipment has the advantages of low cost, relatively simple operation, and being more suitable for routine laboratory analysis. The UHPLC method was successfully applied in the determination of APSs from different growth patterns, species, growth years, and origins of RA. Results showed that Glu was the most abundant monosaccharide, followed by Gal, while the Fuc was the lowest kind. The molar ratio of Gul ranged from 67.48% to 97.76% in the APSs of different RA samples. PCA can accurately distinguish the growth mode of RA and the age of wild-simulated RA based on the monosaccharides contents. In addition, the immunological activity assay demonstrated that APSs could stimulate the NO production in a dose-dependent manner and exhibit a significant correlation with Man, Glu, Xyl, and Fuc contents. Therefore, it can be speculated that the immunological activity of APSs were the result of the joint influence of multiple monosaccharides. Thus, the establishment of a monosaccharides detection UHPLC method in this study can serve as a powerful tool for the constitute analysis of APSs in RA. More importantly, it provides a new perspective tool for qualification of RA medicinal materials and products.

## Figures and Tables

**Table 1:** Detailed information of 109 batches of samples.

Sample No.	Cultivation Patterns	Species	Origin(Province)	Growth Years
S1	Cultivated	*A. membranaceus* var. *mongholicus*	Shanxi	2
S2	Cultivated	*A. membranaceus* var. *mongholicus*	Heilongjiang	2
S3	Cultivated	*A. membranaceus* var. *mongholicus*	Inner Mongolia	1.5
S4–S10	Cultivated	*A. membranaceus* var. *mongholicus*	Inner Mongolia	2
S11	Cultivated	*A. membranaceus* var. *mongholicus*	Inner Mongolia	2.5
S12–S15	Cultivated	*A. membranaceus* var. *mongholicus*	Inner Mongolia	3
S16–S20	Cultivated	*A. membranaceus* var. *mongholicus*	Inner Mongolia	3.5
S21	Cultivated	*A. membranaceus* var. *mongholicus*	Inner Mongolia	4
S22–S24	Cultivated	*A. membranaceus* var. *mongholicus*	Gansu	1
S25	Cultivated	*A. membranaceus* var. *mongholicus*	Gansu	1.5
S26–S39	Cultivated	*A. membranaceus* var. *mongholicus*	Gansu	2
S40–S42	Cultivated	*A. membranaceus* var. *mongholicus*	Gansu	2.5
S43–S45	Cultivated	*A. membranaceus* var. *mongholicus*	Gansu	3
S46–S49	Cultivated	*A. membranaceus* var. *mongholicus*	Gansu	3.5
S50–S51	Cultivated	*A. membranaceus*	Xinjiang	2
S52–S59	Cultivated	*A. membranaceus*	Shanxi	2
S60–S61	Cultivated	*A. membranaceus*	Qinghai	2
S62–S68	Cultivated	*A. membranaceus*	Gansu	2
S69–S71	Wild-simulated	*A. membranaceus* var. *mongholicus*	Shaanxi	1
S72–S74	Wild-simulated	*A. membranaceus* var. *mongholicus*	Shaanxi	2
S75–S77	Wild-simulated	*A. membranaceus* var. *mongholicus*	Shaanxi	3
S78–S81	Wild-simulated	*A. membranaceus* var. *mongholicus*	Shaanxi	4
S82–S84	Wild-simulated	*A. membranaceus* var. *mongholicus*	Shaanxi	5
S85–S91	Wild-simulated	*A. membranaceus* var. *mongholicus*	Shaanxi	6
S92–S99	Wild-simulated	*A. membranaceus* var. *mongholicus*	Shaanxi	7
S100–S104	Wild-simulated	*A. membranaceus* var. *mongholicus*	Shaanxi	8
S105–S106	Wild-simulated	*A. membranaceus* var. *mongholicus*	Shaanxi	9
S107–S109	Wild-simulated	*A. membranaceus* var. *mongholicus*	Shaanxi	10

**Table 2:** Linear relationships of seven monosaccharide derivatives.

Analytes	Linearity			LOD	LOQ
	Equation	R2	Range (μg/mL)	(μg/mL)	(μg/mL)
Mannose	y = 298.99x + 0.6754	0.9995	9.90~396.00	0.66	0.97
Rhamnose	y = 250.85x + 1.1823	0.9977	12.75~510.00	2.55	4.89
Glucose	y = 190.29x + 4.6027	0.9971	641.00~25,665.00	25.67	38.63
Galactose	y = 289.58x + 0.3738	0.9994	50.50~2020.00	11.29	20.20
Arabinose	y = 317.96x + 0.5122	0.9993	22.65~906.00	5.53	9.06
Xylose	y = 427.91x + 0.4024	0.9973	3.85~154.00	7.70	13.60
Fucose	y = 282.39x − 0.0928	0.9987	6.15~246.00	1.23	2.02

**Table 3:** Results of precision, sample recovery, repeatability and stability tests.

Analytes	Precision		Recovery		Repeatability	Stability
	Intra-Day (*n* = 6)	Inter-Day (*n* = 18)	Average Recovery	(*n* = 6)	(*n* = 6)	(*n* = 8)
Mannose	0.87	1.85	99.80	3.45	1.63	1.06
Rhamnose	2.23	3.08	102.45	2.29	3.77	2.69
Glucose	0.53	0.83	95.47	2.38	2.40	0.86
Galactose	0.80	2.67	98.73	3.24	1.97	0.83
Arabinose	1.08	2.72	101.39	4.76	3.18	1.53
Xylose	1.67	4.21	102.16	2.13	2.75	3.23
Fucose	2.48	2.85	103.32	3.38	1.87	4.04

**Table 4:** Comparison of similarity between RA from different sources.

Classification Items	Categories	Similarity
Cultivation patterns	Wild-simulated	0.69~1.00
	Cultivated (Compare with Wild-simulated)	0.84~1.00
Species	MG—2-year-old cultivated RA from Gansu	0.70~1.00
	MJ (Compare with MG)	0.96~1.00
Growth years	2 years—cultivate MG	0.95~1.00
	3 years (Compare with 2 years)	0.74~1.00
	5 and less years—wild-simulated	0.44~0.83
	More than 5 years (Compare with 5 and less years)	0.35~1.00
Origin (province)	Gansu	0.74~1.00
	Inner Mongolia (Compare with Gansu)	0.67~1.00
	Shanxi (Compare with Gansu)	0.95~1.00
	Shaanxi (Compare with Gansu)	0.69~1.00

**Table 5:** Monosaccharide composition of APSs in samples.

Molar Ration (%)							
Test	Mannose	Rhamnose	Glucose	Galactose	Arabinose	Xylose	Fucose
Range (%)	0.10–0.53	0.10–2.42	67.82–97.73	0.95–15.51	0.86–13.99	0.01–1.10	0.02–0.31
Mean (%)	0.52	0.66	87.89	5.30	5.18	0.33	0.11

## Data Availability

The data are available within this article and its Supplementary Materials.

## Supplementary Materials

The following supporting information can be downloaded at: https://www.mdpi.com/article/10.3390/molecules29102287/s1, Table S1: The molar amounts of monosaccharide in 109 batches of samples (mmol/g). Table S2: The molar ration of monosaccharide in 109 batches of samples.

## Abbreviations

RA, Radix Astragali; APS, Astragalus polysaccharides; Man, Mannose; Rha, Rhamnose; Glu, Glucose; Gal, Galactose; Ara, Arabinose; Xyl, Xylose; Fuc, Fucose; UHPLC, Ultra-performance liquid chromatography; PMP, 1-phenyl-3-methyl-5-pyrazolone; NO, Nitric Oxide; TFA, Trifluoroacetic Acid; LOD, limit of detection; LOQ, limit of quantification; RSD, relative standard deviation; PCA, principal component analysis.
