# Supplementary material for: A Rapid and Accurate UHPLC Method for Determination of Monosaccharides in Polysaccharides of Different Sources of Radix Astragali and Its Immune Activity Analysis
Source: Molecules. 2024 May 13;29(10):2287. doi: 10.3390/molecules29102287 (PMC11124152; doi:10.3390/molecules29102287)
Supplement: Supplementary file 1 [file molecules-29-02287-s001.zip › molecules-2981734-supplementary.pdf]

Table S1. The molar amounts of monosaccharide in 109 batches of samples (mmol/g).

Sample.NO	Mannose	Rhamnose	Glucose	Galactose	Arabinose	Xylose	Fucose
S1	0.0128	0.0134	1.4598	0.1142	0.0962	0.0059	0.0030
S2	0.0101	0.0141	1.5666	0.1506	0.1296	0.0067	0.0020
S3	0.0090	0.0136	1.2294	0.1429	0.0775	0.0023	0.0013
S4	0.0125	0.0121	2.3907	0.0863	0.0018	0.0087	0.0036
S5	0.0108	0.0146	2.9096	0.1141	0.1188	0.0103	0.0029
S6	0.0117	0.0148	3.3090	0.0993	0.1024	0.0070	0.0024
S7	0.0081	0.0083	2.4596	0.0790	0.0644	0.0040	0.0014
S8	0.0089	0.0259	0.6952	0.0902	0.1305	0.0014	0.0014
S9	0.0115	0.0145	0.6046	0.1098	0.1214	0.0049	0.0069
S10	0.0080	0.0169	1.0176	0.0915	0.1507	0.0046	0.0018
S11	0.0109	0.0087	1.4793	0.1000	0.1240	0.0101	0.0025
S12	0.0134	0.0175	1.5823	0.1081	0.1592	0.0153	0.0037
S13	0.0068	0.0144	1.9267	0.0874	0.0906	0.0053	0.0012
S14	0.0120	0.0177	4.1610	0.1212	0.1085	0.0071	0.0038
S15	0.0271	0.0281	1.3838	0.2422	0.2239	0.0105	0.0043
S16	0.0082	0.0110	2.4117	0.1130	0.0911	0.0056	0.0018
S17	0.0117	0.0194	1.0923	0.2080	0.1130	0.0106	0.0025
S18	0.0102	0.0177	1.1397	0.1880	0.1124	0.0053	0.0015
S19	0.0091	0.0128	0.8940	0.1668	0.0839	0.0039	0.0019
S20	0.0090	0.0142	1.2253	0.1494	0.0846	0.0079	0.0019
S21	0.0118	0.0136	0.8753	0.0764	0.1605	0.0067	0.0030
S22	0.0066	0.0043	2.7693	0.0429	0.0554	0.0053	0.0018
S23	0.0062	0.0066	2.6896	0.0582	0.0470	0.0020	0.0012
S24	0.0086	0.0067	2.3310	0.0747	0.0576	0.0027	0.0008
S25	0.0096	0.0172	0.9664	0.1518	0.0971	0.0024	0.0018
S26	0.0069	0.0075	1.1373	0.0553	0.0399	0.0007	0.0008
S27	0.0051	0.0097	1.4658	0.1155	0.0606	0.0028	0.0009
S28	0.0091	0.0162	1.3539	0.1537	0.0989	0.0054	0.0017
S29	0.0198	0.0269	2.5921	0.2654	0.1772	0.0140	0.0050
S30	0.0119	0.0197	1.7830	0.1480	0.1432	0.0069	0.0028
S31	0.0086	0.0106	1.5665	0.0868	0.0790	0.0047	0.0014
S32	0.0136	0.0149	1.2632	0.1238	0.0992	0.0043	0.0026
S33	0.0099	0.0128	1.4430	0.1247	0.0884	0.0049	0.0018
S34	0.0087	0.0109	3.0485	0.1262	0.0767	0.0044	0.0008
S35	0.0135	0.0188	1.0061	0.2649	0.1563	0.0066	0.0020
S36	0.0082	0.0114	1.3418	0.1277	0.0743	0.0037	0.0016
S37	0.0052	0.0024	2.3802	0.0416	0.0430	0.0039	0.0006

S38	0.0067	0.0087	1.9973	0.0675	0.0728	0.0035	0.0011
S39	0.0048	0.0051	3.9492	0.0385	0.0371	0.0042	0.0020
S40	0.0090	0.0121	1.3106	0.0810	0.0805	0.0025	0.0017
S41	0.0047	0.0075	0.9517	0.0541	0.0470	0.0036	0.0008
S42	0.0081	0.0127	1.2581	0.0978	0.0907	0.0032	0.0017
S43	0.0135	0.0163	3.5384	0.1218	0.1398	0.0114	0.0036
S44	0.0090	0.0118	2.5059	0.1307	0.1390	0.0087	0.0017
S45	0.0120	0.0194	4.4777	0.1194	0.0945	0.0088	0.0014
S46	0.0090	0.0095	2.2107	0.1050	0.0894	0.0048	0.0016
S47	0.0088	0.0127	1.1218	0.0886	0.0815	0.0031	0.0015
S48	0.0107	0.0152	0.8602	0.1916	0.1532	0.0040	0.0007
S49	0.0089	0.0122	1.2302	0.1022	0.0882	0.0038	0.0017
S50	0.0106	0.0067	2.0308	0.0612	0.0860	0.0030	0.0014
S51	0.0117	0.0198	1.4110	0.0794	0.1704	0.0054	0.0026
S52	0.0112	0.0125	1.0054	0.1659	0.1118	0.0037	0.0016
S53	0.0082	0.0105	1.8973	0.0957	0.0683	0.0006	0.0009
S54	0.0083	0.0083	2.6607	0.0980	0.0783	0.0026	0.0017
S55	0.0094	0.0093	1.9218	0.1187	0.0904	0.0047	0.0014
S56	0.0102	0.0137	1.8148	0.1161	0.1086	0.0084	0.0020
S57	0.0107	0.0110	1.9420	0.1161	0.0905	0.0042	0.0016
S58	0.0051	0.0082	1.1923	0.0634	0.0640	0.0060	0.0011
S59	0.0099	0.0098	3.1800	0.0757	0.0760	0.0076	0.0029
S60	0.0139	0.0153	1.8662	0.1452	0.1063	0.0052	0.0030
S61	0.0090	0.0122	1.4464	0.1457	0.0844	0.0036	0.0018
S62	0.0057	0.0063	1.3823	0.0523	0.0419	0.0009	0.0005
S63	0.0149	0.0139	1.5137	0.1320	0.1318	0.0100	0.0027
S64	0.0054	0.0051	1.2554	0.0637	0.0491	0.0000	0.0015
S65	0.0099	0.0099	1.8131	0.0875	0.1052	0.0036	0.0013
S66	0.0081	0.0107	1.4864	0.0585	0.0778	0.0032	0.0014
S67	0.0196	0.0105	1.0900	0.0601	0.1228	0.0089	0.0036
S68	0.0074	0.0139	1.3164	0.0552	0.0651	0.0026	0.0013
S69	0.0239	0.0293	1.8916	0.1493	0.2207	0.0169	0.0056
S70	0.0077	0.0114	3.0790	0.0823	0.0980	0.0098	0.0027
S71	0.0018	0.0019	0.4628	0.0247	0.0227	0.0023	0.0005
S72	0.0086	0.0135	1.9244	0.0730	0.0702	0.0097	0.0017
S73	0.0085	0.0107	1.5281	0.0816	0.1344	0.0091	0.0023
S74	0.0107	0.0141	2.5730	0.0890	0.1069	0.0084	0.0024
S75	0.0061	0.0084	3.5975	0.0632	0.0837	0.0118	0.0021
S76	0.0082	0.0101	3.5786	0.0511	0.1451	0.0080	0.0030
S77	0.0080	0.0118	3.5180	0.0822	0.1138	0.0111	0.0033
S78	0.0091	0.0117	2.1057	0.0809	0.1278	0.0121	0.0015
S79	0.0173	0.0227	1.7536	0.1638	0.2041	0.0175	0.0052
S80	0.0100	0.0134	4.0119	0.0934	0.1123	0.0096	0.0021
S81	0.0170	0.0163	2.9375	0.1230	0.1713	0.0110	0.0045

S82	0.0114	0.0118	2.5198	0.0982	0.1185	0.0101	0.0018
S83	0.0083	0.0080	1.7709	0.0638	0.0863	0.0070	0.0014
S84	0.0140	0.0184	1.7796	0.1407	0.1800	0.0108	0.0028
S85	0.0451	0.0523	1.2491	0.2449	0.2648	0.0275	0.0097
S86	0.0103	0.0127	2.4160	0.0799	0.1131	0.0090	0.0021
S87	0.0101	0.0101	1.8421	0.0816	0.1007	0.0099	0.0030
S88	0.0075	0.0092	2.1270	0.0859	0.0696	0.0054	0.0014
S89	0.0056	0.0064	2.5033	0.0616	0.0667	0.0029	0.0011
S90	0.0085	0.0067	2.6435	0.0695	0.0715	0.0063	0.0015
S91	0.0209	0.0246	2.0743	0.1013	0.1057	0.0101	0.0047
S92	0.0069	0.0121	3.1221	0.0663	0.0886	0.0070	0.0023
S93	0.0096	0.0103	2.5169	0.0716	0.1043	0.0063	0.0019
S94	0.0140	0.0162	2.1936	0.1163	0.1591	0.0113	0.0032
S95	0.0072	0.0102	3.4440	0.0699	0.0840	0.0069	0.0026
S96	0.0108	0.0141	2.5838	0.1077	0.1418	0.0115	0.0022
S97	0.0068	0.0097	4.3476	0.0702	0.1018	0.0099	0.0017
S98	0.0120	0.0169	2.5770	0.1238	0.1409	0.0127	0.0074
S99	0.0044	0.0080	4.3283	0.0434	0.0381	0.0066	0.0009
S100	0.0103	0.0121	2.3334	0.0889	0.1028	0.0067	0.0020
S101	0.0099	0.0089	2.3056	0.1005	0.1005	0.0111	0.0019
S102	0.0074	0.0096	2.9467	0.0740	0.0906	0.0113	0.0026
S103	0.0140	0.0157	1.1683	0.0809	0.1066	0.0155	0.0035
S104	0.0115	0.0207	2.5115	0.0948	0.1136	0.0090	0.0028
S105	0.0109	0.0142	1.7264	0.0867	0.1198	0.0101	0.0044
S106	0.0196	0.0214	3.0824	0.1711	0.1655	0.0113	0.0029
S107	0.0200	0.0197	1.9930	0.1242	0.1532	0.0134	0.0054
S108	0.0148	0.0145	3.3005	0.0875	0.0946	0.0120	0.0038
S109	0.0159	0.0099	1.7521	0.1011	0.1009	0.0041	0.0020

Table S2. The molar ration of monosaccharide in 109 batches of samples.

Sample.NO	Mannose	Rhamnose	Glucose	Galactose	Arabinose	Xylose	Fucose
S1	0.0075	0.0079	0.8560	0.0670	0.0564	0.0035	0.0018
S2	0.0054	0.0075	0.8334	0.0801	0.0690	0.0036	0.0011
S3	0.0061	0.0092	0.8330	0.0968	0.0525	0.0016	0.0009
S4	0.0050	0.0048	0.9503	0.0343	0.0007	0.0035	0.0014
S5	0.0034	0.0046	0.9146	0.0359	0.0374	0.0032	0.0009
S6	0.0033	0.0042	0.9330	0.0280	0.0289	0.0020	0.0007
S7	0.0031	0.0032	0.9371	0.0301	0.0245	0.0015	0.0005
S8	0.0095	0.0169	0.7371	0.0957	0.1384	0.0010	0.0015
S9	0.0142	0.0243	0.6782	0.1338	0.1365	0.0098	0.0031
S10	0.0052	0.0066	0.8789	0.05338	0.0517	0.0033	0.0011
S11	0.0063	0.0050	0.8524	0.0576	0.0714	0.0058	0.0014
S12	0.0070	0.0092	0.8330	0.0569	0.0838	0.0081	0.0020

S13	0.0032	0.0068	0.9036	0.0410	0.0425	0.0025	0.0005
S14	0.0027	0.0040	0.9390	0.0274	0.0245	0.0016	0.0009
S15	0.0141	0.0146	0.7208	0.1262	0.1166	0.0055	0.0022
S16	0.0031	0.0042	0.9127	0.0428	0.0345	0.0021	0.0007
S17	0.0081	0.0133	0.7495	0.1427	0.0775	0.0073	0.0017
S18	0.0069	0.0120	0.7728	0.1275	0.0762	0.0036	0.0010
S19	0.0077	0.0109	0.7625	0.1423	0.0716	0.0034	0.0016
S20	0.0060	0.0095	0.8211	0.1001	0.0567	0.0053	0.0013
S21	0.0103	0.0119	0.7629	0.0666	0.1399	0.0058	0.0026
S22	0.0023	0.0015	0.9597	0.0149	0.0192	0.0018	0.0006
S23	0.0022	0.0024	0.9569	0.0207	0.0167	0.0007	0.0004
S24	0.0035	0.0027	0.9392	0.0301	0.0232	0.0011	0.0003
S25	0.0077	0.0138	0.7753	0.1218	0.0779	0.0020	0.0014
S26	0.0056	0.0060	0.9109	0.0443	0.0320	0.0005	0.0006
S27	0.0031	0.0059	0.8828	0.0695	0.0365	0.0017	0.0006
S28	0.0056	0.0099	0.8261	0.0938	0.0603	0.0033	0.0010
S29	0.0064	0.0087	0.8360	0.0856	0.0572	0.0045	0.0016
S30	0.0056	0.0093	0.8428	0.0700	0.0677	0.0033	0.0013
S31	0.0049	0.0060	0.8913	0.0494	0.0449	0.0027	0.0008
S32	0.0089	0.0098	0.8302	0.0814	0.0652	0.0028	0.0017
S33	0.0059	0.0076	0.8561	0.0740	0.0524	0.0029	0.0011
S34	0.0027	0.0033	0.9305	0.0385	0.0234	0.0013	0.0002
S35	0.0098	0.0137	0.7353	0.1205	0.1142	0.0049	0.0015
S36	0.0052	0.0073	0.8553	0.0814	0.0474	0.0023	0.0011
S37	0.0021	0.0010	0.9610	0.0168	0.0173	0.0016	0.0002
S38	0.0031	0.0040	0.9257	0.0313	0.0337	0.0016	0.0005
S39	0.0012	0.0013	0.9773	0.0095	0.0092	0.0010	0.0005
S40	0.0060	0.0081	0.8753	0.0541	0.0537	0.0017	0.0011
S41	0.0044	0.0070	0.8899	0.0506	0.0439	0.0034	0.0008
S42	0.0055	0.0086	0.8545	0.0664	0.0616	0.0022	0.0012
S43	0.0035	0.0043	0.9203	0.0317	0.0364	0.0030	0.0009
S44	0.0032	0.0042	0.8928	0.0466	0.0495	0.0031	0.0006
S45	0.0025	0.0041	0.9460	0.0252	0.0200	0.0019	0.0003
S46	0.0037	0.0039	0.9097	0.0432	0.0368	0.0020	0.0006
S47	0.0067	0.0096	0.8512	0.0672	0.0618	0.0024	0.0011
S48	0.0086	0.0123	0.6962	0.1551	0.1240	0.0033	0.0005
S49	0.0062	0.0085	0.8500	0.0706	0.0609	0.0027	0.0012
S50	0.0048	0.0030	0.9232	0.0278	0.0391	0.0013	0.0006
S51	0.0069	0.0116	0.8299	0.0467	0.1002	0.0032	0.0015
S52	0.0086	0.0095	0.7662	0.1264	0.0852	0.0028	0.0012
S53	0.0039	0.0050	0.9115	0.0460	0.0328	0.0003	0.0004
S54	0.0029	0.0029	0.9310	0.0343	0.0274	0.0009	0.0006
S55	0.0044	0.0043	0.8915	0.0551	0.0419	0.0022	0.0006
S56	0.0049	0.0066	0.8751	0.0560	0.0524	0.0040	0.0010

S57	0.0049	0.0051	0.8924	0.0533	0.0416	0.0019	0.0007
S58	0.0038	0.0061	0.8898	0.0473	0.0478	0.0044	0.0008
S59	0.0030	0.0029	0.9459	0.0225	0.0226	0.0022	0.0009
S60	0.0064	0.0071	0.8659	0.0674	0.0493	0.0024	0.0014
S61	0.0053	0.0072	0.8493	0.0855	0.0495	0.0021	0.0011
S62	0.0038	0.0042	0.9278	0.0351	0.0281	0.0006	0.0003
S63	0.0082	0.0076	0.8322	0.0726	0.0724	0.0055	0.0015
S64	0.0039	0.0037	0.9096	0.0462	0.0356	0.0000	0.0011
S65	0.0049	0.0049	0.8929	0.0431	0.0518	0.0018	0.0006
S66	0.0049	0.0065	0.9030	0.0356	0.0473	0.0019	0.0008
S67	0.0149	0.0080	0.8285	0.0457	0.0934	0.0068	0.0028
S68	0.0062	0.0131	0.7882	0.0709	0.1168	0.0035	0.0014
S69	0.0102	0.0126	0.8093	0.0639	0.0944	0.0072	0.0024
S70	0.0023	0.0035	0.9356	0.0250	0.0298	0.0030	0.0008
S71	0.0034	0.0037	0.8956	0.0479	0.0440	0.0044	0.0010
S72	0.0041	0.0064	0.9159	0.0348	0.0334	0.0046	0.0008
S73	0.0048	0.0060	0.8611	0.0460	0.0757	0.0051	0.0013
S74	0.0038	0.0050	0.9174	0.0317	0.0381	0.0030	0.0009
S75	0.0016	0.0022	0.9535	0.0168	0.0222	0.0031	0.0006
S76	0.0022	0.0027	0.9407	0.0134	0.0381	0.0021	0.0008
S77	0.0021	0.0032	0.9386	0.0219	0.0304	0.0030	0.0009
S78	0.0039	0.0050	0.8964	0.0345	0.0544	0.0051	0.0006
S79	0.0079	0.0104	0.8028	0.0750	0.0935	0.0080	0.0024
S80	0.0023	0.0032	0.9434	0.0220	0.0264	0.0023	0.0005
S81	0.0052	0.0050	0.8954	0.0375	0.0522	0.0034	0.0014
S82	0.0041	0.0043	0.9091	0.0354	0.0427	0.0037	0.0007
S83	0.0043	0.0041	0.9102	0.0328	0.0444	0.0036	0.0007
S84	0.0065	0.0086	0.8291	0.0656	0.0839	0.0050	0.0013
S85	0.0153	0.0197	0.7628	0.0885	0.1007	0.0107	0.0023
S86	0.0039	0.0048	0.9141	0.0302	0.0428	0.0034	0.0008
S87	0.0049	0.0049	0.8953	0.0396	0.0489	0.0048	0.0014
S88	0.0032	0.0040	0.9224	0.0373	0.0302	0.0023	0.0006
S89	0.0021	0.0024	0.9456	0.0233	0.0252	0.0011	0.0004
S90	0.0030	0.0024	0.9416	0.0248	0.0255	0.0022	0.0005
S91	0.0089	0.0105	0.8858	0.0433	0.0451	0.0043	0.0020
S92	0.0021	0.0037	0.9446	0.0201	0.0268	0.0021	0.0007
S93	0.0035	0.0038	0.9250	0.0263	0.0383	0.0023	0.0007
S94	0.0056	0.0065	0.8726	0.0463	0.0633	0.0045	0.0013
S95	0.0020	0.0028	0.9501	0.0193	0.0232	0.0019	0.0007
S96	0.0038	0.0049	0.8996	0.0375	0.0494	0.0040	0.0008
S97	0.0015	0.0021	0.9560	0.0154	0.0224	0.0022	0.0004
S98	0.0042	0.0058	0.8915	0.0428	0.0487	0.0044	0.0026
S99	0.0010	0.0018	0.9771	0.0098	0.0086	0.0015	0.0002
S100	0.0040	0.0047	0.9129	0.0348	0.0402	0.0026	0.0008

S101	0.0039	0.0035	0.9083	0.0396	0.0396	0.0044	0.0007
S102	0.0024	0.0030	0.9378	0.0236	0.0288	0.0036	0.0008
S103	0.0100	0.0112	0.8318	0.0576	0.0759	0.0110	0.0025
S104	0.0041	0.0075	0.9087	0.0343	0.0411	0.0032	0.0010
S105	0.0055	0.0072	0.8752	0.0440	0.0607	0.0051	0.0022
S106	0.0056	0.0062	0.8872	0.0493	0.0476	0.0033	0.0008
S107	0.0086	0.0084	0.8558	0.0533	0.0658	0.0057	0.0023
S108	0.0042	0.0041	0.9356	0.0248	0.0268	0.0034	0.0011
S109	0.0080	0.0050	0.8822	0.0509	0.0508	0.0021	0.0010
